# Structural basis of periplasmic bridge protein docking and nucleotide-dependent remodelling in lipopolysaccharide transport

**DOI:** 10.21203/rs.3.rs-10942018/v1

**Published:** 2026-10-15

**Authors:** Jani Bolla, Yi Wang, Matteo Cervoni, Hayden Tucker, Keyi Wei, Corinne Lutomski, Tarick El-Baba, Francesco Imperi, Phillip Stansfeld, Carol Robinson, Francesco Fiorentino, Chih-Chia Su

## Abstract

The outer membrane of Gram-negative bacteria is assembled and maintained by the lipopolysaccharide (LPS) transport machinery, which extracts LPS from the inner membrane and conveys it across the periplasm via a protein bridge. How the inner-membrane transporter, LptB _2_ FGC, engages the soluble bridge component, LptA (named LptH in *Pseudomonas aeruginosa* ), and how this junction responds to the ATPase cycle remain unresolved. Here, we determine cryo-electron microscopy structures of the *P. aeruginosa* LptB _2_ FGC-LptH complex in nucleotide-free and AMP-PNP-bound states at 3.24 Å and 3.05 Å resolution, respectively. The structures show that LptH augments the terminal beta-strand of LptC, thereby completing a continuous beta-jellyroll conduit. ATP analogue binding closes the LptB dimer and the transmembrane LPS-binding cavity while remodelling the LptF-LptC junction at the base of the assembled bridge. Molecular dynamics simulations show that the LptC-LptH connection remains intact but becomes less compact in the ATP-bound ensemble. Native mass spectrometry further reveals that thanatin preferentially disrupts LptH-LptH and LptC-LptH assemblies. Together, these findings establish the architecture of the transporter-bridge junction and reveal how nucleotide-driven closure remodels an already assembled periplasmic conduit while preserving bridge continuity.

